# The Choice of an Anesthetic for Children1Read at the thirty-fourth annual meeting of the Medical Association of Central New York, held at Buffalo, N. Y., August 27 and 28, 1901.

**Published:** 1901-10

**Authors:** Edward Judson Wynkoop

**Affiliations:** Syracuse, N. Y.


### The Choice of an Anesthetic for Children.[1]

By EDWARD JUDSON WYNKOOP, Syracuse, N. Y.

IN GOING over the literature of the past few years the reference to anesthetics occurs with increasing frequency, showing that the interest in this important branch is beginning to receive its deserved attention. Nothing so thoroughly shocks the profession and laity as a death during anesthesia, and to avoid this calamity, every physician and surgeon strives. That deaths during anesthesia do and will occur, we all realise, but if greater care can be taken in the choice of an anesthetic anesthetiser, and in the physical examination, at least, the death-roll might be lessened.

In the choice of anesthetics for children many things are to be considered, and the old saying, "chloroform for pregnant women and children, ether for adults," will bear further investigation. The most popular anesthetic for children today is unquestionably chloroform.

In speaking of anesthetics, this paper will consider simply four, which probably today are the most in use in this country, namely—chloroform, ether, nitrous oxide gas, and bromide of ethyl.

For some time past various committees have been appointed to determine the action of the different anesthetics. The reports of the Hyderabad Commission, which pointed out that chloroform kills through paralysis of respiration, and occasionally through paralysis of the heart, is no longer accepted, and even its chairman, Lauder Brunton[1], says, "that if we drive chloroform

1. Read at the thirty-fourth annual meeting of the Medical Association of Central New York, held at Buffalo, N. Y., August 27 and 28, 1901.

into the trachea or air into the lungs, loaded with chloroform, it will be absorbed in sufficient quantity to paralyse the heart." Wood[2] states, "that this is a practical giving away of the whole case of the Hyderabad Commission, at least, as I have understood it, and I believe it to have been understood by the general profession." More recent investigations[3] have proven conclusively that chloroform is a depressant to the circulation, and more often kills by paralysis of the heart than by paralysis of respiration. Chloroform is direct poison to the heart muscle, and by its action reduces blood pressure.

According to Hare, the cause of death is often caused by vaso-motor paralysis, allowing the patient to bleed to death in his own blood-vessels[4]. The irritant effect of chloroform upon the kidneys is less marked than ether, although transitory albuminuria[5] is quite likely to occur even when no evidence of albumin was present before the narcosis. It does not, as a rule, irritate the throat and lungs, if properly given, and vomiting is an unusual occurrence when proper attention has been given the stomach of the patient. Except, then, for its action on the heart and respiratory center, chloroform is an ideal anesthetic, but, knowing as we do this action on the heart, are we justified in its routine use? Ether, which is a well-known cardiac stimulant[6] has some disadvantages. It requires more of the anesthetic to get a patient narcotised. By some it is claimed that it is a marked irritant to the throat, lungs and kidneys, and liable to provoke vomiting.

Bromide of ethyl is a cardiac depressant resembling chloroform[7] in its action, causing muscular rigidity[8] and a tendency towards hemorrhage.[9] It is liable to impurities and it has no advantage over chloroform.

Nitrous oxide gas is probably more often used than any other anesthetic, and owing to its physiological action, death is not common. The objection to its use lies in its very short stage of anesthesia, allowing only the shortest possible operation to be done. It has been proven that the cause of this anesthesia is due to asphyxia[10]. Statistics offered through Dr. George M. Gould,[11] of Philadelphia and the Lancet Commission, prove conclusively that deaths during chloroform anesthesia occur more often than when ether is used. For instance: 638,461 administrations of chloroform anesthesia, a total of 160 deaths; 300,175 administrations of ether, with a total of 18 deaths; chloroform mortality, 1 to 3,749; ether mortality, 1 to 16,675.

Though nitrous oxide gas is far more safe than ether and chloroform, yet the stage of complete anesthesia is so short, its effect in operative work is slight; however, in statistics given, when nitrous oxide gas proceeded the use of ether, the death rate was even less than with ether alone,[12] yet today many physicians prefer chloroform to ether.

P. Wagner[13] gives the record of deaths as follows: Pentall, 1 in 199; chloroform, 1 in 2,900; chloroform and ether, 1 in 4,100; bromide of ethyl, 1 in 450; ether, 1 in 15,500.

The report of the German Surgical Society[14] shows the proportion of deaths from chloroform much greater than from ether The statistics everywhere obtainable, both in this country and other countries, show the mortality from chloroform to exceed that from ether. Statistics of this country are somewhat difficult to obtain, but those which can be found show a decided mortality from chloroform and less from ether.[15]

Abbe[25] in 1895 stated that in his experience, kidney and lung complications following ether are usually due to other causes than the ether and in children he invariably used ether.

About one year ago Wyeth[16] gave as his experience, that in children under twelve, ether was far preferable to chloroform. About this time Hinkel[17] and Halstead[18] reported their observations in children with the lymphatic diathesis, and advised the use of ether in this condition.

In brief review of the few points of the physiological action of these four anesthetics, it would seem that ether is the safest anesthetic in general surgery. Exact records in regard to anesthetics administered to children are hard to obtain, and we only have the statistics as a whole to aid us. Our consideration, then, is to choose some anesthetic which, from its physiological action, is least dangerous, and which in every way is easy in administering and reliable in its effects. We know chloroform is many times more powerful in its depressing action on the heart than ether, therefore, why use it? In children with the lymphatic diathesis it is, without doubt, dangerous.

Edmond Owen[19] states that it is sad to have to admit that fatal accidents in connection with the administration of chloroform, even in the case of children, are occurring with increasing frequency. He further states that there is always risk in giving chloroform or any other anesthetic to a child. In this same paper he reports two deaths from chloroform. One in a child operated on for harelip; the other for post-nasal growth, neither of these two cases occurring in his practice, however. The use of ether in throat and mouth operations is often objected to on account of the irritation produced, but if a small amount of atropin precede the ether and the ether given properly, but little need be feared from these secretions.

In pulmonary troubles there is reason for giving chloroform, but if serious cardiac weakness is also present, it would seem to me that ether with oxygen or preceded by nitrous oxide gas would be better.

In regard to heart disease, the recent paper of Finney, of Johns Hopkins[20] proves very instructive. I cannot do better than quote from this able article. "As a rule, in all cases of weak heart from whatever cause, chloroform is the most dangerous. It is more dangerous too in very nervous, apprehensive patients, in kidney diseases, and the like, while ether in acute lung affections and alcoholics is more dangerous probably than chloroform."

His report of 142 cases is very interesting. It would seem, then, that the all important point is the condition of the heart muscle, and not so much the nature of the valvular lesion.

I cannot refrain from quoting the closing passages of this article:

I cannot emphasise too strongly my conviction that in severe operations the anesthetist plays a most important, and in some cases a more important rôle than the operator, and one of the reforms most urgently needed in the medical profession of our country today is a thoroughly competent corps of anesthetists in our hospitals and in our medical schools, and a thorough and complete course of instruction in the proper methods of administration and use of these agents so powerful for good when rightfully used, and so useful for relief in suffering humanity and yet capable of producing such disastrous results.

Stengel[21] states that "in myocardial diseases under ether anesthesia, these patients show an improved cardiac condition. Myocarditis is the condition which the surgeon has reason to fear."[22] Hare[23] considers ether by far the safest anesthetic, if properly administered, excepting in vascular diseases. He gives its contraindications "high arterial tension, due to vascular change and grave vascular disease." But he prefers ether in Bright's disease, if proper precautions are taken.. Atropin he considers of great value in these conditions and should always be used. These reforms hold true for children as well as adults.

In regard to the action of ether on the air-passages, Holscher, of Kiel,[24] gives the result of his studies as follows: (1) Aside from slight increase of mucous secretions, ether vapor has no other irritant action on the tracheo-bronchial mucous membrane; (2) that the tracheal gargle of ether narcosis is a result of the aspiration of the mouth secretions, and can be avoided by proper technique: Attention to the face, exit of the mouth secretions by lowering and turning of the head and elevating or separating of the angle of the lips. Attention to the maintenance of free respiration by holding the lower jaw forward and preventing the tongue from falling back into the tracheal space; (3) the affection of the air-passages following ether narcosis are usually the result of the aspiration of infected mouth contents; (4) the glistening appearance of the bronchial and tracheal epithelium is not altered during narcosis; (5) the increased salivary secretions during ether narcosis, although chiefly due to the narcosis are not entirely the result of local irritation of the ether vapors; more often there is also a central action.

In a series of experiments on animals Thompson and Kemp[28] show that ether causes some damage to the kidneys, and they believe it to be contraindicated in renal disease. However the general result of experiments showing the effect of ether on the kidneys of man and animals proves the damage much less than formerly supposed.

Ogden[26] reports a series of observations and remarks that the urine of children did not show evidence of any more marked renal disturbance from ether than that of adults.

Weir[27] believes ether safer when the kidneys are even slightly affected. His conclusions are as follows: (1) Ether narcosis produces no perceptible effect on the healthy kidneys in man or animals; (2) it is not dangerous in healthy, nor when the kidneys are only slightly diseased; (3) any effects it may produce on the kidneys are only transient.

In the foregoing lines the attempt has been made to show that ether can be used as an anesthetic for children and that the results of the investigations today show it can be used more and with greater safety than other anesthetics.

The combination of ether and oxygen[29] or ether and gas[30] are no doubt of great aid, but ether alone, properly given has few contraindications.

So many articles have been published in regard to proper administration of ether that it seems useless to discuss it here, but the tendency is toward improved methods of administration of anesthetics and the thrusting of these important agents into skilled hands.

1. Dennis's System of Surgery, Vol. I., p. 652.
2. " " " " I., p. 652.
3. " " " " I., p. 650.
4. H. A. Hare. Therapeutic Gazette, February, 1897.
5. Sajous's Annual and Analytical Cyclopedia, Vol. II., p. 151, 152.
6. H. C. Wood. Therapeutics, p. 140, 141.
Hare. Practical Therapeutics, p. 161.
White-Wilcox, p. 241.
7. Sajous's Annual and Analytical Cyclopedia, Vol. II., p. 2.
8. " " " " " II., p. 2.
9. " " " " " II., p. 2.
10. " " " " " V., p. 210.
11. H. C. Wood. Dennis's System of Surgery, Vol. I., p. 646.
12. " " " " " " " I., p. 646.
13. Sajous's Annual and Analytical Cyclopedia, Vol. III., p. 214.
14. " " " " " III., p. 206.
15. " " " " " III., p. 206.
16. Wyeth. Medical Record, March 10, 1900.
17. Halsted. Philadelphia Medical Journal, November 3, 1900.
18. Hinkel. New York Medical Journal, October 29, 1895.
19. Owen. Archives of Pediatrics, March, 1895.
20. Finney. American Journal of Medical Sciences, August, 1901.
21. Stengel. " " " " " "
22. Mayo. " " " " " "
23. Hare. " " " " " "
24. Holscher. Medical Record, January 28, 1899.
25. Abbe. " " June 8, 1895.
26. Ogden. New York Medical Journal, July 25, 1897.
27. Weir. " " " " November 16, 1895.
28. Thompson and Kemp. Medical Record, September 3, 1898.
29. Cole. Medical Record, October 12, 1895.
30. Golden. Journal of the American Medical Association, December 15, 1900.

### DISCUSSION.

Dr. A. L. Beahan, Canandaigua.—I think Dr. Wynkoop has written a very fair paper in regard to the question of the use of our common anesthetics, especially ether and chloroform. There is one point that occurs to me and that is, when we are speaking of the dangers of anesthesia we are always directing our attention to the condition of the kidneys. If we had a preliminary examination of the kidneys, in a large number of cases we would find disease. This is always fair in a discussion of the question of the use of ether as an anesthetic, and it being our American anesthetic we should be loyal to it and not discard it because of the reputation it has in its effect upon the kidneys; whereas anyone can give chloroform, it is an easy anesthetic to administer, but it is dangerous in the early stages of anesthesia, and it should be discarded because of that and should receive the disapproval of men working, especially in the larger operations. Of course, in the operations upon children it has its use.

Dr. S. L. Snow, Syracuse.—I have listened with a good deal of interest to this paper, as the doctor tries to emphasize here the importance of a still anesthetic. As I work more and more I am impressed with this fact, that we need a still anesthetic. As to one point he made in regard to bromide of ethyl I would take issue with him. He says that bromide of ethyl increases hemorrhage. That may be so in major operations, but in the short operations that are necessary in nose and throat work I find that it does not increase hemorrhage. There is much less hemorrhage in the use of bromide of ethyl than there is in ether. It is very common for me to be seriously interrupted when I use ether in the removal of adenoids by the flow of blood, and I find now that in using bromide of ethyl the flow of blood is insignificant, particularly if the patient is in the upright position. I suppose I am departing perhaps from the usual rule, but I often use bromide of ethyl for the removal of adenoids, and I feel quite safe in administering it in the upright position. Chloroform I always thought a little carefully about. The bromide of ethyl, I predict, is an anesthetic that will be very popular and come into common use with throat specialists. Dr. Morganthrau, of Chicago, I think, has collected 200 cases of his own. Dr. Dresden, of New York, recently read a paper before an association at New Haven. I think he has collected 500 cases where he has operated with no untoward results. Dr. Hollenbeck, of Chicago, has operated a great many times, one or two hundred; another specialist in St. Louis a large number, and we are getting reports in the journals right along of the men who are using bromide of ethyl for these operations, and they are all very favorable. I myself have used it more than fifty times, and each time with perfect success and with no untoward results. If you will allow me just a moment, I will say that the bromide of ethyl should always be procured in steel tubes. I use none but Merck's. I insist on those tubes coming to me in the original package, wrapped up, from the laboratory. I think that the drug is very unsafe if used from the bottle. I think it should be in hermetically sealed tubes, wrapped in blue paper. I have always insisted on this and I have never had any unpleasant results. It can be administered like ether. The patient breathes it one minute; that is my rule. Some of them go under within forty seconds. Adults, I often find, have to take it one minute and drop right off; go to sleep. I will tell you of an instance. Dr. Belknap happened in the operating room the other day in the Hospital of the Good Shepherd and he asked what anesthetic I was using. I said bromide of ethyl. He picked up the anesthetic and placed it to his nose, and then started to go to the window to show me his automobile. I looked around in a moment and saw him reaching for a chair. He had not breathed it more than fifteen seconds before he wanted to sit down. I have repeatedly removed tonsils and adenoids, the whole operation, from the beginning of the anesthetic to the end of the anesthetic, not taking more than two minutes. That is very rapid work, though; three minutes usually covers the time the patient is under the anesthetic. When they come out from the influence of the anesthetic they have their faculties and they can spit out the blood. If I was going to have a patient under the anesthetic more than five minutes I should use ether, but in these slight operations, like removal of adenoids and tonsils, it is a very fine thing.

Dr. Wm. B. Jones, Rochester.—I only heard a part of the paper, and that related to chloroform. First of all, the more dangerous anesthetic is the one which a person is not used to, unless the man using it is very skilled. Anyone who sees a great deal of anesthesia will say that the anesthesia occasions some little anxiety, and it is easy to vary a little the method of administration, or even the drug itself, to prevent symptoms of which there are warnings. These symptoms, in my experience, come oftener with ether than with chloroform. I have seen things which were attributed to anesthesia, when in fact the difficulty occurred before the administration of the anesthetic, or some time after its effect had passed away. One patient I saw died on the table before the anesthetic was given. As I read the reports of deaths from anesthetics, the deaths from anesthesia in nearly every case are considered to be those which occur upon the table, and although the after-effect of the anesthetic is mentioned, yet I have never yet seen a tabulated report or any comparisons between the deaths from chloroform and those from ether which included those that were due to the after-effects. My own observation gives me a proportion of about five to one in favor of chloroform. I have never seen but one death from anesthetics in which there was not a warning, and a warning sufficient to enable an expert to prevent death; and therefore I firmly think that all the deaths reported from ether or chloroform, occurring within an hour from the end of the operation, that they are due to the physician's method of giving the anesthetic, or else to some exigency of the operation which made it necessary to give the anesthetic even after it was shown to be dangerous. The remarks upon bromide of ethyl were interesting, and if the doctor would say a word about his own impressions on the drug, I think it would be still more satisfactory.

Dr. Snow.—I have an impression that is growing upon me that bromide of ethyl is far safer than other drugs, if it is used rightly and with the right kind of cone. I do not think it is safe to take it away from the face and reapply it again, and I do not think it is safe to use the drug after the bottle has been opened or the tube has been opened. I always take one tube and break it and put it into the cone, and the next time I use another tube, and I feel much less concern about these patients than I did when I was giving chloroform. I have used it now about two years, and I am much pleased with it. If I had a child of my own and it had to have the tonsils or adenoids removed, I should expect it to be comparatively safe, because so far we have been unable to discover any particular change in the pulse, and the face is not flushed; the patients want to sit up, immediately when they awake, and you can wake them quickly, have their full faculties and they are able to sit up; they are perfectly strong, able to do anything. One little fellow was given a few whiffs of it and he dropped off to sleep so nicely that we thought he was all right, and I put the instrument in and he came out as quick as a flash. In that case we had to do our work very quickly. We did not hurt him any, though he woke up and began to struggle. We give them the anesthetic forty or fifty seconds and they are sound asleep, and you do the work quickly and say, "spit out the blood," and they wake up and spit it out. The patient is only under from a minute and a half to three minutes, and it seems to me that that is an element of safety.

Dr. Wynkoop.--I wish to say a word in regard to the effect of ether upon the kidneys. I think that investigation along that line has shown that wherever there has been any abnormal effect upon the kidneys or upon any of the organs it is due to improper use of the ether, and in the use of the gas the results are much better. My paper was simply to call attention to the use of ether in children, and I believe we can use it a great deal more in children than we do. That was the object of the paper.

